# The Bonn-Risk-Index for determination of the risk of urolith formation and other metabolic diseases

**DOI:** 10.1186/1878-5085-5-S1-A63

**Published:** 2014-02-11

**Authors:** Norbert Laube, Wolfgang Berg, Thomas Knoll, Heinz Busch

**Affiliations:** 1 NTTF Coatings GmbH, D-53619 Rheinbreitbach, Germany; 2 Klinik und Poliklinik für Urologie, Universitätsklinikum Jena, D-07743 Jena, Germany; 3 Klinikum Sindelfingen-Böblingen, Sindelfingen, D-71065 Sindelfingen, Germany

## Scientific objectives

The Bonn-Risk-Index (BRI) for the determination of the urinary stone formation risk is based on a crystallization test induced within a sample of native urine. The established method shows superior diagnostic sensitivity and specificity to other risk indices, derived from the urinary concentrations of some selected major urinary constituents considered to be indicators of the urine’s supersaturation with respect to the mineral phase formed by the patient. However, urinary supersaturation, the major thermodynamic driving force for crystal formation, is governed by all – organic and inorganic – components coexisting in the solution. In addition to the individual concentration of the components their mutual interactions also play an important role. Thus, BRI which “simulates” stone formation within whole urine meets much better the analytical demands to describe the “true supersaturation” than the costly determination of a couple of individual parameters. Indeed, minor constituents may take higher influence on an induced crystallisation process as one might expect from its urinary concentration. Urine composition reflects the influence of any of the patient’s metabolic processes – even of those analytically not directly accessible. Therefore, a change in urine composition leading to a variation in stone formation risk can indicate a change in the patient’s general metabolic state. Thus, BRI can also be used to monitor the general metabolic state by taking into account all urinary components, even unknown and difficult to access ones. In particular, changes in the calcium metabolisms could be detected as BRI is especially developed for the evaluation of the risk of formation of calcium stones. Clinical studies that systematically investigate the BRI, for example, in patients with bone diseases, like osteoporosis or Paget’s Disease, or in patients with diseases, which still lack a quantifiable progression parameter, should be run.

## Outlook

In case of clinically relevant correlations between BRI and the severity and course of a metabolic disease BRI can be used to monitor this disease. Therefore, BRI and the BRI-on-Chip rapid test under development could be cost-effective diagnostic tools for many other metabolic diseases than urolithiasis, too.

